# Unravelling the triad of penicillin-binding proteins, β-lactamase activity, and mRNA dynamics in *Pseudomonas aeruginosa* AmpC induction

**DOI:** 10.1093/jac/dkaf408

**Published:** 2025-11-05

**Authors:** Maria Montaner, Mariella Montes, Francina Alajarin, Silvia López-Argüello, Antonio Oliver, Bartolome Moya

**Affiliations:** Health Research Institute of the Balearic Islands (IdISBa), Palma, Spain; Health Research Institute of the Balearic Islands (IdISBa), Palma, Spain; Health Research Institute of the Balearic Islands (IdISBa), Palma, Spain; Health Research Institute of the Balearic Islands (IdISBa), Palma, Spain; Health Research Institute of the Balearic Islands (IdISBa), Palma, Spain; Servicio de Microbiología, Hospital Universitario Son Espases, Palma, Spain; Centro de Investigación Biomédica en Red en Enfermedades Infecciosas (CIBERINFEC), Madrid, Spain; Health Research Institute of the Balearic Islands (IdISBa), Palma, Spain; Centro de Investigación Biomédica en Red en Enfermedades Infecciosas (CIBERINFEC), Madrid, Spain

## Abstract

**Objectives:**

The main objective of the present work was to dissect the interplay between penicillin-binding protein (PBP) occupancy, *ampC* transcriptional dynamics, and β-lactamase activity in *Pseudomonas aeruginosa*.

**Methods:**

Using wild-type PAO1 and isogenic LMW-PBP knockout mutants (PAOΔ*dacB*, PAOΔ*dacBdacC*, and PAOΔ*dacBdacCpbpG*), we assessed *ampC* induction following exposure to 18 β-lactams and β-lactamase inhibitors. PBP binding (IC_50_) was quantified via Bocillin-FL labelling. AmpC mRNA levels were measured by qRT-PCR, and β-lactamase activity was determined in crude extracts, periplasmic and extracellular fractions using nitrocefin and cefalotin substrates.

**Results:**

Carbapenems and cefoxitin, the drugs that maximally inhibited PBP4, induced strong *ampC* transcription and β-lactamase activity, while ceftazidime, aztreonam, and penicillins (PBP3-binding) triggered mRNA upregulation without enzymatic output. Furthermore, LMW-PBP-deficient mutants showed stepwise *ampC* overexpression (up to ∼7000-fold), but activity increases plateaued after ∼1100-fold, even after a significant mRNA induction following incubation with ceftazidime.

**Conclusions:**

These findings highlight that *ampC* transcription does not predict functional enzyme levels, especially for PBP3-binding drugs. Full induction appears to require PBP4 targeting and CreBC activation. These findings underscore the need for integrated assessment of PBP IC_50_, transcription, and enzymatic activity to guide rational β-lactam and β-lactamase inhibitor therapy, and lay the groundwork for future studies on alternative *ampC* regulatory pathways.

## Introduction

*Pseudomonas aeruginosa* remains a formidable clinical challenge due to its intrinsic resistance mechanisms, among which the upregulation of the chromosomal inducible cephalosporinase-type AmpC β-lactamase [*Pseudomonas*-derived β-lactamase (PDC)] stands as a pivotal contributor to multidrug resistance.^1^ The dynamic interplay between beta-lactam antibiotics and AmpC expression underscores the complexity of bacterial adaptive responses, highlighting the need for a comprehensive understanding of the molecular mechanisms driving this phenomenon.^2^

Additionally, most *P. aeruginosa* strains possess the genes for OXA-50 and PIB-1 β-lactamases. Despite the widespread distribution of PIB-1, its expression in the laboratory strain PAO1 and in clinical isolates is negligible. OXA-50, also known as PoxB, has been acknowledged as a naturally occurring oxacillinase in this bacterial species. However, neither of these enzymes has been experimentally linked to β-lactam resistance, which is primarily attributed to the presence of the AmpC cephalosporinase.^3–5^

In *P. aeruginosa*, the regulation of *ampC* is tightly controlled and inducible in response to certain β-lactam antibiotics. Central to the regulation of the *ampC* beta-lactamase in *P. aeruginosa* are the penicillin-binding proteins (PBPs), which serve as the primary targets of beta-lactam antibiotics. PBPs are essential enzymes involved in peptidoglycan biosynthesis and play a key role in maintaining cell wall integrity.^2,6^ During active growth, the bacterial cell wall undergoes constant remodelling, requiring controlled cleavage of peptidoglycan by dedicated hydrolases to permit insertion of new material and successful cell division. Although a portion of the resulting fragments is released into the extracellular milieu, the majority are transported back into the cytoplasm via the AmpG permease as part of the recycling pathway.^2,7^ Once internalized, the muropeptides [predominantly *N*-acetylglucosamine-1,6-anhydro-*N*-acetylmuramyl-peptides (NAG-anhNAM-peptides)] are sequentially processed: NagZ removes the terminal NAG, and AmpD hydrolyses the bond linking the 1,6-anhNAM to the peptide stem.^2,7–9^ This process liberates sugars and peptides that are reincorporated into peptidoglycan biosynthesis, ultimately contributing to the generation of UDP-*N*-acetylmuramyl-pentapeptide (UDP-NAM-P5), a critical precursor. Under basal conditions, UDP-NAM-P5 binds to the transcriptional regulator AmpR in a configuration that represses *ampC* expression, thus maintaining β-lactamase production at minimal levels in the absence of cell wall stress.^2^

However, exposure to inducer β-lactams, such as cefoxitin, disrupts the equilibrium of peptidoglycan turnover favouring autolysis by inhibiting LMW-PBPs, notably PBP4.^2,8^ The disruption of peptidoglycan recycling leads to the saturation of NagZ and AmpD leading to the transient accumulation of cell wall degradation products such as anhMurNAc-pentapeptides (anhNAM-P5). These molecules interact with the LysR-type transcriptional regulator AmpR, converting it from a repressor to an activator of *ampC* transcription.^2,6,10,11^ Mutations in *ampD* or to a lower extent related amidases (*ampDh2*, *ampDh3*) can lead to constitutive hyperexpression of *ampC* and β-lactam resistance.^8,12^

Furthermore, inactivation of the LMW-PBPs, PBP5/6 (*dacC*), PBP7 (*pbpG*), and particularly PBP4 (*dacB*), results in the constitutive accumulation of the cell wall degradation products which in turn lead to high-level β-lactam resistance.^2,6,10,11^ PBP4 inhibition and deletion have been shown to cause the activation of the CreBC two-component system in contrast with AmpD (AmpDh2 and AmpDh3) disruption, which has no effect on this system.^2,13,14^ The abnormal buildup of anhNAM-P5 following PBP4 inactivation acts as a key trigger activating CreBC through CreC sensor while also boosting *ampC* expression via AmpR. This over-accumulation of soluble anhNAM-P5 likely underlies the role of PBP4 as a cell wall stress sentinel, acting as the signal sensed by the membrane-bound CreC sensor. This, in turn, triggers CreBC activation and simultaneously promotes *ampC* expression through AmpR binding.^2,8,10,11,15,16^

In *P. aeruginosa*, *ampC* mRNA levels and β-lactamase activity are related but not interchangeable measures of resistance. Quantifying *ampC* transcripts gives an early readout of gene induction, often before any detectable enzymatic activity, but doesn’t always translate to functional resistance due to possible post-transcriptional or post-translational regulation.^6,11,17^ On the other hand, β-lactamase activity assays directly measure the hydrolysis of β-lactams, providing a functional snapshot, but they’re influenced by growth phase and metabolic state and can miss early low-level activity. Furthermore, post-transcriptional mechanisms, such as mRNA stability and translation efficiency, further modulate beta-lactamase expression and contribute to the overall antibiotic resistance phenotype.^15,18^ Relying on mRNA alone risks overlooking the actual resistance phenotype, while enzyme assays may not catch early shifts. Using both methods in parallel gives a more accurate picture of resistance progression in clinical isolates.^7,11,12,19–21^

It is essential to evaluate the actual inducibility potential of β-lactams, as their classification as either strong or weak *ampC* inducers significantly influences their likelihood of selecting for mutations that result in *ampC* constitutive derepression.^6,17,22^ Understanding the underlying determinants of *ampC* differential regulation is crucial for predicting antibiotic resistance selection and optimizing therapeutic interventions.

In this manuscript, we aimed to unravel the triad of PBP-mediated signalling, mRNA dynamics, and β-lactamase activity underlying *P. aeruginosa* AmpC induction in wild-type PAO1 and its isogenic mutants of LMW-PBPs. By leveraging a multidisciplinary approach that integrates genetic, biochemical, and gene expression analyses, we seek to expand our understanding on the factors that influence AmpC expression dynamics.

## Materials and methods

### Bacterial strains and *in vitro* susceptibility testing

*P. aeruginosa* PAO1 and its isogenic LMW-PBPs knockouts, PAOΔ*dacB*, PAOΔ*dacBdacC*, and PAOΔ*dacBdacCpbpG* (which our group previously documented as constitutively hyper-expressing AmpC), were utilized in this study.^11^ MICs for imipenem, doripenem, meropenem, ertapenem, cefepime, ceftazidime, cefoxitin, aztreonam, piperacillin, ticarcillin, carbenicillin, mecillinam, and the β-lactamase inhibitors avibactam, clavulanic acid, relebactam, tazobactam, and sulbactam (all from MedChem Express, Sollentuna, Sweden) were determined by Clinical and Laboratory Standards Institute (CLSI) broth microdilution.^23^

### PBP binding assays

Binding to PBP2, PBP3, PBP4, and PBP5 was quantified in intact PAO1 at 30, 90, and 180 min after exposure to twelve β-lactams (four carbapenems, three cephalosporins, three antipseudomonal penicillins, one monobactam) plus four β-lactamase inhibitors. Overnight 100 mL LB cultures were harvested (4000 rpm, 20 min, RT), washed four times in PBS, and finally resuspended to 2 mL. Aliquots (150 µL) were dispensed into microtiter plates and exposed to 2-fold antibiotic dilutions. PBP labelling with Bocillin-FL and IC_50_ calculation followed the protocol previously described.^13,14^

### Induction of AmpC expression

Cultures of PAO1 and its LMW-PBPs knockouts (PAOΔ*dacB*, PAOΔ*dacBdacC*, PAOΔ*dacBdacCpbpG*) were grown in 30 mL Mueller Hinton broth (1:100 inoculum from overnight cultures) and incubated for 3 h at 37°C, 180 rpm with each drug at 0.5 × MIC (a dose that triggers AmpC signalling and avoids excessive killing during the 180-min activity assessment) and at the concentration that half maximally inhibited PBP4 after 30 min exposure (PBP4_30_IC_50_). Untreated controls ran in parallel. At 30, 90, and 180 min, 3 mL aliquots were harvested (4000 rpm, 15 min, 4°C), supernatants were discarded, and cell pellets were collected. Each pellet was then split: one half for total RNA isolation and qRT-PCR, the other for protein extraction and β-lactamase assays as detailed below.

### Determination of *ampC* expression through quantitative reverse transcription polymerase chain reaction (qRT-PCR)

Total RNA was extracted with the RNeasy Mini Kit (Qiagen, Hilden, Germany), treated with TURBO DNase (Ambion, Austin, TX, USA), and quantified on a NanoDrop One (Thermo Fisher Scientific, Waltham, MA, USA). Normalized (50 ng/mL) RNA aliquots were subjected to one-step reverse transcription and qPCR with the QuantiTect SYBR Green kit (Qiagen) on a CFX Connect thermocycler (Bio-Rad, Hercules, CA, USA). Primer pairs ACrnaF/ACrnaR (*ampC*) and rpsL-1/rpsL-2 (housekeeping ribosomal protein S12; *rpsL*) were used as previously reported.^16^ Relative expression was calculated by the 2^−ΔΔCt^ method from at least two biological and two technical replicates, normalized to each strain’s t = 0 min baseline.^24^

### Assessment of the specific enzymatic activity (SEA) of the β-lactamase AmpC

The specific enzymatic activity (SEA) of AmpC was quantified as nmol nitrocefin hydrolysed min^−1^ mg^−1^ protein by monitoring A_482_ in the presence of 10^−5^ M nitrocefin, exactly as described previously.^25^ Proteins were harvested from three compartments: crude extract, periplasm, and culture supernatant, and activities were expressed relative to the untreated PAO1 (or mutant) sample at t = 0 min.^16^ To confirm that hydrolysis was not substrate-dependent, parallel assays were run with cefalotin (10 mM, ΔA_260_). AmpC specificity was verified by pre-incubating crude extracts with cloxacillin at concentrations of 50 and 100 μM (equivalent to approximately 23.8 and 47.6 mg/L, respectively).^16,25,26^ Periplasmic fractions were prepared by lysozyme/MgCl_2_ spheroplasting as in our earlier work;^27^ extracellular activity was measured directly in cell-free supernatants collected at the same time points.^28^

### Statistical data analysis

The GraphPad Prism software (version v9.01) was used for graphical representation and statistics. One-way ANOVA with *post hoc* Tukey’s multiple comparison tests was run to determine the statistically significant differences over time. Unpaired Student’s *t*-tests were applied to determine statistically significant differences between mRNA, qRT-PCR, and SEA determinations (Table S1, available as Supplementary data at *JAC* Online).

## Results

### Differential induction patterns among carbapenems, cephalosporins, and penicillins

**Figure 1.**
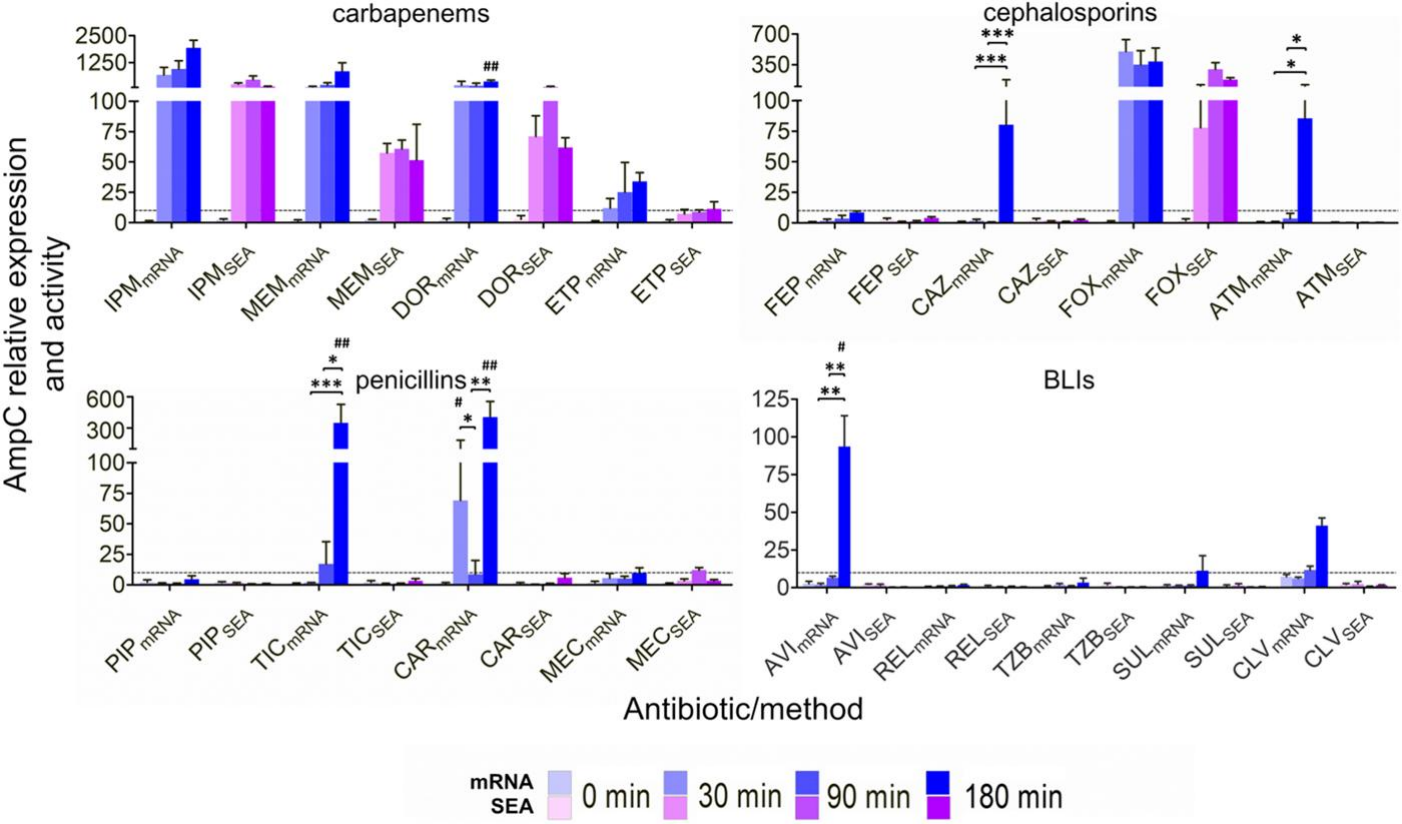
The relative expression (baseline measurements at t = 0 min) of *ampC* (mRNA) in *P. aeruginosa* strain PAO1, as measured by qRT-PCR, and the corresponding relative specific enzymatic activity (SEA) were assessed following exposure to 0.5 × MIC of various β-lactams and β-lactamase inhibitors at time points of 0, 30, and 180 min. Results are averages of at least two sets (biological replicates) of two technical replicates. One-way ANOVA with *post hoc* Tukey’s multiple comparison test was run to determine the statistically significant differences over time (*P < 0.05; **P < 0.01; ***P < 0.005). Unpaired Student’s *t*-tests were applied to determine statistically significant differences between mRNA and SEA determinations (^#^*P* < 0.05; ^##^*P* < 0.01; ^###^*P* < 0.001). Drug abbreviations are detailed in Table 1, while the concentrations employed are defined in Table 2.

At 0.5 × MIC (Table 1), carbapenems were the most potent *ampC* inducers. Imipenem drove a 700-fold rise in transcripts within 30 min and achieved higher expression thereafter (1900-fold at 180 min); meropenem and doripenem produced similar profiles peaking at 875- and 377-fold, respectively, whereas ertapenem triggered only minimal transcription. Relative mRNA and enzymatic outputs tracked closely for all four drugs. Cefoxitin, another strong inducer, sustained parallel increases in *ampC* transcripts (385–500-fold) and β-lactamase activity (77–296-fold), while cefepime caused virtually no response. Ceftazidime and aztreonam each produced a late, ∼80-fold jump in mRNA at 180 min, yet neither yielded detectable enzyme activity. Piperacillin and mecillinam were poor inducers, but ticarcillin and carbenicillin boosted transcripts 347- and 404-fold, respectively, again without measurable activity (Figure 1).

**Table 1.** Susceptibility data and concentrations used in the AmpC induction experiments for 18 β-lactam antibiotics and BLIs in intact cells of P. aeruginosa PAO1

β-Lactam subclass	Drug	MIC (mg/L)^a^	Studied drug concentration in 0.5 × MIC induction assays (mg/L)	Studied drug concentration in PBP4_30_IC_50_ induction assays (mg/L)^b^
Carbapenem	Imipenem	1	0.5	0.01
Carbapenem	Doripenem	1	0.5	0.02
Carbapenem	Meropenem	0.5	0.25	0.02
Carbapenem	Ertapenem	4	2	0.05
Penicillin	Piperacillin	4	2	1.67
Penicillin	Carbenicillin	32	16	4.98
Penicillin	Ticarcillin	16	8	5.21
Penicillin	Mecillinam	16	8	211.78
Monobactam	Aztreonam	4	2	1.35
Cephalosporin	Ceftazidime	1	0.5	0.32
Cephalosporin	Cefepime	1	0.5	0.25
Cephalosporin	Cefoxitin	1024	512	20.42
DBO-BLI	Avibactam	ND	4	2.39
DBO-BLI	Relebactam	ND	4	8.49
BLI	Tazobactam	ND	4	4.78
BLI	Sulbactam	ND	4	2.89
BLI	Clavulanic Acid	ND	4	4.34

IC_50_ after 30 min incubation as determined in Table 2.

^a^Broth microdilution MICs were performed following CLSI guidelines.^23^

^b^Concentrations obtained from the PBP IC_50_ experiments, concentration equal to PBP4.

Among β-lactamase inhibitors, only clavulanic acid and avibactam raised *ampC* mRNA, and this transcriptional signal did not translate into increased enzyme output.

### Correlating PBP4 inhibition with *ampC* induction: mRNA levels and enzymatic activity in classic inducers

Binding to PBP4 is a recognized trigger for *ampC* induction. Canonical inducers, cefoxitin, imipenem, and the BLIs clavulanic acid and avibactam, all promote *ampC* transcription via this pathway. To test whether a partial PBP4 occupancy (50% binding at 30 min, PBP4_30_IC_50_; Table 1) elicited comparable responses, we repeated the induction assays at these lower concentrations (Figure S1 and Table S2) following prior determination of the IC_50_ values for PBP2, PBP3, PBP4, and PBP5/6 (Table 2). Carbapenem IC_50_ values were 12–50-fold below 0.5 × MIC; penicillins were 1.5–5-fold lower (mecillinam required 35-fold more); cephalosporins were 1.5–2-fold lower, except cefoxitin (25-fold lower); β-lactamase inhibitor (BLI) values varied ±2-fold from the standard 4 mg/L (Table 1).

**Table 2.** PBP binding (IC_50_) of β-lactam antibiotics and BLIs in whole cells of *P. aeruginosa* PAO1 assessed following incubation periods of 30, 90, and 180 min

Time (min)	PBP		Drug mg/L^a^							
		IPM	ETP	MEM	DOR	PIP	TIC	CAR	MEC	ATM
30	PBP2	0.06 ± 0.02	0.32 ± 0.04	1.53 ± 0.38	0.29 ± 0.05	2.18 ± 0.54	19.8 ± 3.25	32.3 ± 12.6	283 ± 65.2	7.42 ± 6.70
	PBP3	1.01 ± 0.64	0.54 ± 0.32	0.60 ± 0.26	0.44 ± 0.12	**0.88** ± **0.25**	**2.46** ± **1.13**	**3.41** ± **2.57**	564 ± 71.3	**0.76** ± **0.44**
	PBP4	**0.01** ± **0.004**	**0.05** ± **0.001**	**0.02** ± **0.01**	**0.02** ± **0.01**	1.67 ± 0.67	4.98 ± 2.02	5.21 ± 5.13	**211** ± **82.1**	1.35 ± 0.72
	PBP5	0.06 ± 0.02	0.81 ± 0.12	1.40 ± 0.22	0.90 ± 0.25	3.15 ± 2.11	262 ± 66.1	48.8 ± 26.9	423 ± 226	4.55 ± 3.84
90	PBP2	0.07 ± 0.01	0.07 ± 0.04	0.14 ± 0.08	0.36 ± 0.17	1.54 ± 0.30	11.9 ± 7.85	24.4 ± 7.81	58.2 ± 11.8	6.52 ± 2.07
	PBP3	1.51 ± 0.63	0.25 ± 0.16	0.32 ± 0.12	0.22 ± 0.15	**0.78** ± **0.25**	**2.24** ± **1.18**	7.27 ± 3.57	95.1 ± 33.7	**0.26** ± **0.07**
	PBP4	**0.01** ± **0.005**	**0.03** ± **0.01**	**0.01** ± **0.001**	**0.02** ± **0.01**	0.64 ± 0.25	3.19 ± 0.22	**3.04** ± **0.92**	**35.1** ± **2.86**	1.41 ± 1.51
	PBP5	0.14 ± 0.01	0.80 ± 0.24	0.68 ± 0.16	1.01 ± 0.19	6.35 ± 11.6	166 ± 89.4	48.8 ± 26.9	376 ± 118	7.03 ± 4.19
180	PBP2	0.15 ± 0.04	0.26 ± 0.11	0.18 ± 0.03	0.05 ± 0.04	2.13 ± 0.52	14.21 ± 4.72	13.67	**24.2** ± **3.15**	1.32 ± 0.32
	PBP3	0.93 ± 0.22	0.10 ± 0.04	2.24 ± 0.76	0.64 ± 0.27	**0.49** ± **0.28**	**2.92** ± **1.88**	**3.32** ± **2.22**	80.8 ± 32.8	**0.16** ± **0.05**
	PBP4	**0.01** ± **0.005**	**0.02** ± **0.01**	**0.01** ± **0.001**	**0.01** ± **0.005**	0.38 ± 0.14	3.93 ± 1.39	3.87 ± 0.73	39.2 ± 4.45	1.22 ± 0.62
	PBP5	0.12 ± 0.07	0.73 ± 0.31	0.48 ± 0.27	0.33 ± 0.11	0.79 ± 0.87	68.7 ± 14.8	21.3 ± 18.2	254 ± 157	24.8 ± 1.37

		FEP	CAZ	FOX	AVI	CLV	REL	TZB	SUL
30	PBP2	0.96 ± 0.37	1.49 ± 0.26	2029 ± 834	6.29 ± 2.33	11.2 ± 2.59	5.95 ± 3.86	**3.58** ± **2.81**	4.48 ± 3.19
	PBP3	**0.21** ± **0.07**	**0.31** ± **0.24**	2303 ± 932	10.3 ± 3.25	6.07 ± 3.79	**4.25** ± **0.59**	4.02 ± 1.52	2.12 ± 0.86
	PBP4	0.25 ± 0.05	0.32 ± 0.06	20.4 ± 1.64	**2.39** ± **0.28**	4.34 ± 3.02	8.49 ± 7.58	4.78 ± 4.84	2.89 ± 0.36
	PBP5	4.36 ± 3.70	21.1 ± 12.1	**5.31** ± **2.18**	3.28 ± 1.11	**3.60** ± **1.48**	28.46 ± 26.57	4.76 ± 1.11	**2.09** ± **0.83**
90	PBP2	0.52 ± 0.12	2.96 ± 1.58	749 ± 235	4.20 ± 2.32	5.19 ± 0.82	2.58 ± 1.84	2.04 ± 0.51	5.95 ± 1.77
	PBP3	**0.25** ± **0.04**	**0.35** ± **0.26**	1618 ± 848	3.51 ± 1.54	3.56 ± 1.55	4.76 ± 2.79	**0.95** ± **0.77**	8.37 ± 2.05
	PBP4	0.63 ± 0.22	2.14 ± 1.98	14.5 ± 7.3	**1.17** ± **0.16**	**2.38** ± **1.18**	**2.00** ± **1.14**	1.60 ± 0.47	3.42 ± 1.88
	PBP5	1.07 ± 1.69	12.2 ± 15.02	**4.15** ± **0.45**	1.27 ± 0.13	2.47 ± 1.72	2.86 ± 1.89	1.88 ± 0.59	**1.68** ± **1.45**
180	PBP2	1.12 ± 0.57	1.38 ± 0.27	52.9 ± 37.4	2.82 ± 2.06	20.3 ± 5.39	11.5 ± 2.44	2.14 ± 1.71	2.88 ± 1.14
	PBP3	**1.46** ± **0.68**	**0.21** ± **0.04**	406 ± 190	6.90 ± 2.92	3.12 ± 0.57	8.12 ± 6.41	3.34 ± 1.77	7.52 ± 4.12
	PBP4	0.21 ± 0.08	1.01 ± 0.82	5.24 ± 2.56	**1.12** ± **0.24**	**2.50** ± **1.87**	**2.05** ± **0.13**	2.15 ± 0.90	2.15 ± 1.15
	PBP5	1.08 ± 2.19	11.5 ± 5.02	**3.38** ± **1.80**	1.71 ± 0.37	9.27 ± 3.22	2.68 ± 0.22	**2.07** ± **1.06**	**1.24** ± **0.70**

Bold letters represent the most inhibited PBP.

IPM, imipenem; ETP, ertapenem; MEM, meropenem; DOR, doripenem; PIP, piperacillin; TIC, ticarcillin; CAR, carbenicillin; MEC, mecillinam; ATM, aztreonam; FEP, cefepime; CAZ, ceftazidime; FOX, cefoxitin; AVI, avibactam; REL, relebactam; TZB, tazobactam; SUL, sulbactam; CLV, clavulanic acid; ZID, zidebactam.

^a^Concentration of drug that inhibits 50% of the Bocillin-FL signal compared to that of a control containing no drug.

**Figure 2.**
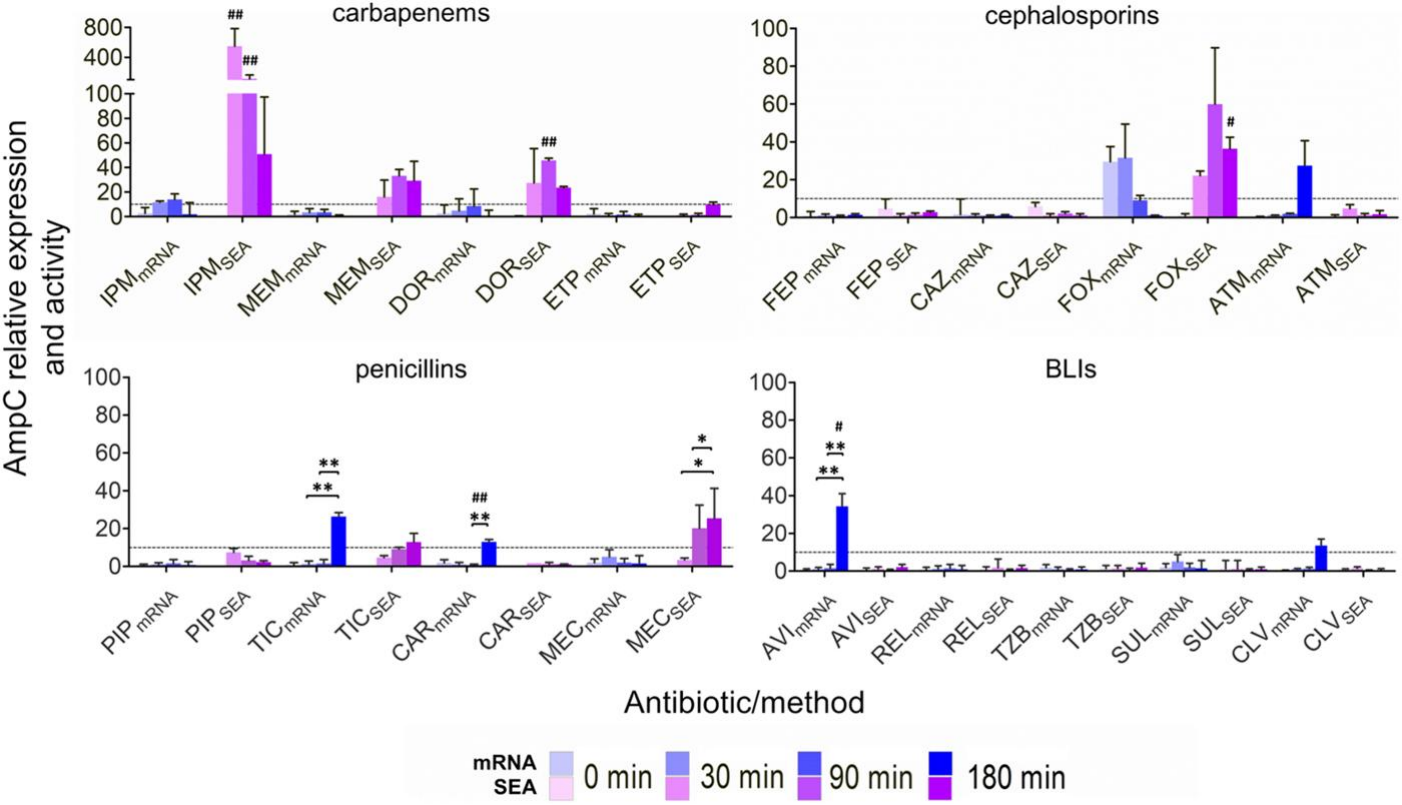
Relative *ampC* qRT-PCR (mRNA) expression and relative specific enzymatic activity (SEA) in *P. aeruginosa* strain PAO1 after 0, 30, and 180 min exposure to the concentration that half maximally inhibits PBP4 after 30 min (PBP4_30_IC_50_) of drug exposure of different β-lactams and β-lactamase inhibitors relative to basal (ctrl; time = 0 min). Results are averages of at least two sets (biological replicates) of two technical replicates. One-way ANOVA with *post hoc* Tukey’s multiple comparison test was run to determine the statistically significant differences over time (*P< 0.05; **P< 0.01; ***P< 0.005). Unpaired Student’s *t*-tests were applied to determine statistically significant differences between mRNA and SEA determinations (^#^*P*< 0.05; ^##^*P*< 0.01; ^###^*P*< 0.001). Drug abbreviations are detailed in Table 1, while the concentrations employed are defined in Table 2.

Under PBP4 IC_50_ conditions, *ampC* mRNA induction remained below the 10-fold threshold for most drugs; only cefoxitin, aztreonam, ticarcillin, carbenicillin, avibactam, and clavulanic acid exceeded this threshold (Figure 2). However, enzyme output exhibited higher significant changes (Table S1): canonical inducers imipenem and cefoxitin boosted AmpC peak activity ∼550-fold and ∼50-fold, 30 and 90 min post-exposure, respectively, while meropenem, doripenem, and mecillinam produced 20–45-fold increases at 90 min and ∼25-fold at 180 min.

### Limited *ampC* inducibility in LMW-PBP-deficient mutants

Our investigation into the binding of antibiotics to PBP4 and its role in *ampC* induction in PAO1 prompted us to examine the contribution of all the LMW-PBPs when fully inhibited. To this end, we performed parallel experiments using isogenic knockout mutants PAOΔ*dacB*, PAOΔ*dacBdacC*, and PAOΔ*dacBdacCpbpG.*^11^

**Figure 3.**
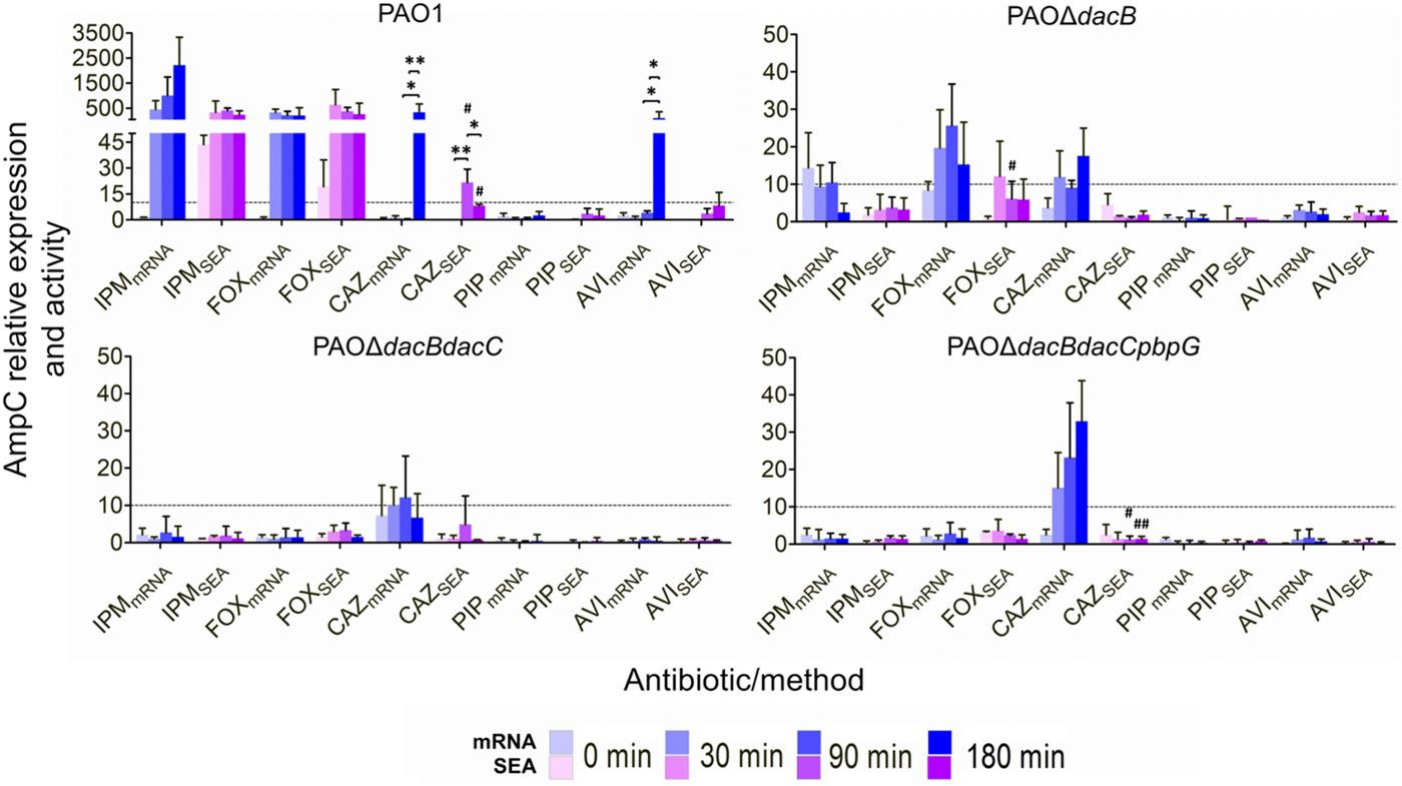
The relative expression (baseline measurements of each strain at t = 0 min) of *ampC* (mRNA) in *P. aeruginosa* strains PAO1 and isogenic mutants PAOΔ*dacB*, PAOΔ*dacBdacC*, and PAOΔ*dacBdacCpbpG* as measured by qRT-PCR, and the corresponding relative specific enzymatic activity (SEA) were measured after exposing the strains to 0.5 × MIC of various β-lactams and β-lactamase inhibitors at intervals of 0, 30, and 180 min. Results are averages of at least two sets (biological replicates) of two technical replicates. One-way ANOVA with *post hoc* Tukey’s multiple comparison test was run to determine the statistically significant differences over time (*P < 0.05; **P < 0.01; ***P < 0.005). Unpaired Student’s *t*-tests were applied to determine statistically significant differences between mRNA and SEA determinations (^#^*P* < 0.05; ^##^*P* < 0.01; ^###^*P* < 0.001). Drug abbreviations are detailed in Table 1, while the concentrations employed are defined in Table 2.

Sequential inactivation of the LMW-PBP set (PAOΔ*dacB*, PAOΔ*dacBdacC*, and PAOΔ*dacBdacCpbpG*) drove a stepwise rise in constitutive *ampC* expression of 24-, 1500-, and 7000-fold above PAO1, respectively, yet relative AmpC activity climbed more modestly (24-, 902-, and 1154-fold) (Figure S2). SEA peaked at 1521 pmol nitrocefin min^−1^ mg^−1^ of protein in the triple mutant after 3 h. However, further inducibility proved limited: only the single mutant showed modest mRNA boosts with cefoxitin, imipenem, or ceftazidime. Although ceftazidime (PBP-3 binding; Table 2 and Table S2) further increased transcripts in the double and triple mutants, the enzyme activity remained unaltered (Figure 3).

**Figure 4.**
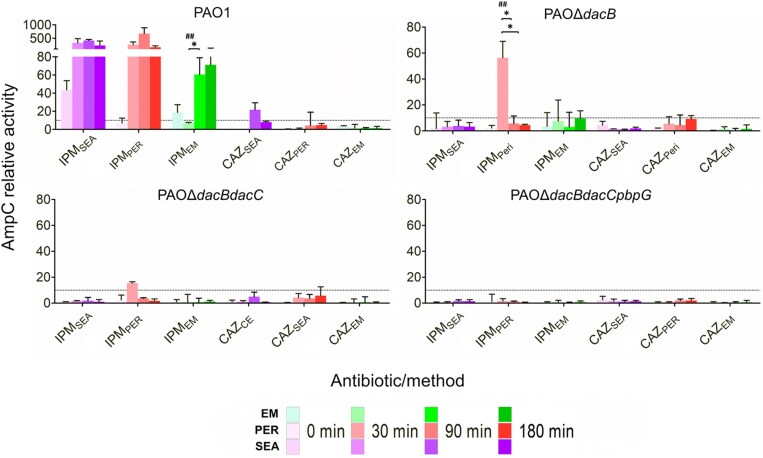
The relative specific enzymatic activity of different cellular compartments [crude extract (SEA), periplasmic (PER), and extracellular medium (EM)] of AmpC in *P. aeruginosa* strains PAO1 and isogenic mutants, PAOΔ*dacB*, PAOΔ*dacBdacC*, and PAΔ*dacBdacCpbpG*, was assessed after treatment with 0.5 × MIC (per each strain) of different β-lactams and β-lactamase inhibitors. Measurements were taken at the time intervals of 0, 30, and 180 min. Results are averages of at least two sets (biological replicates) of two technical replicates. One-way ANOVA with *post hoc* Tukey’s multiple comparison test was run to determine the statistically significant differences over time (*P < 0.05; **P < 0.01; ***P < 0.005). Unpaired Student’s *t*-tests were applied to determine statistically significant differences between mRNA and SEA determinations (^#^*P* < 0.05; ^##^*P* < 0.01; ^###^*P* < 0.001). Drug abbreviations are detailed in Table 1, while the concentrations employed are defined in Table 2.

To exclude sample-handling losses as the cause of the mismatch between *ampC* transcripts and β-lactamase output under ceftazidime treatment (PBP3-binding, filamentation), we determined activity in supernatant, periplasm, and crude extract after ceftazidime incubation, using imipenem (PBP2-binding, spheroplast formation) as a comparator. In PAO1, imipenem drove extracellular, crude extract, and periplasmic activities up 20–60-, 416-, and 637-fold, respectively, yet no fraction of the double or triple mutants registered any differential value after imipenem or ceftazidime exposure (Figure 4).

To confirm that our enzyme readings were not conditioned by the use of nitrocefin as substrate, we repeated the inductions in PAO1 with imipenem, cefoxitin, and ticarcillin (0.5 × MIC and PBP4_30_IC_50_) using cefalotin as an alternative substrate (Table S3). Specific enzymatic activities (SEA) with cefalotin closely tracked those obtained with nitrocefin throughout the 180 min time course, showing only minor point-to-point variation. Thus, AmpC activity proved substrate-independent under all conditions tested. As before, ceftazidime failed to elicit activity in any LMW-PBP knockout, regardless of substrate. Finally, adding 500 mg/L cloxacillin to crude extracts of the triple mutant (PAOΔ*dacBdacCpbpG*), which constitutively overproduces AmpC, nearly abolished activity with both substrates (>95% reduction in the hydrolysis rates), confirming that the results obtained were AmpC-specific (Table S4).

## Discussion

Understanding the regulatory dynamics of *ampC* expression in *P. aeruginosa* is essential to anticipate emerging β-lactam resistance. In this study, we investigate the relationship between *ampC* transcriptional activity and β-lactamase production across a representative panel of β-lactam and β-lactamase inhibitors, with a particular focus on the differential roles of PBPs.

The differential induction patterns observed among carbapenems, cephalosporins, and penicillins underscore the complexity of *ampC* regulation in response to structurally distinct β-lactams. While carbapenems such as imipenem and meropenem consistently triggered robust *ampC* transcription accompanied by measurable β-lactamase activity, the lack of enzymatic response—despite pronounced mRNA upregulation—observed with late-phase inducers like ceftazidime and aztreonam suggests the involvement of distinct regulatory pathways that remain to be elucidated.^16,29^ A similar dissociation between transcriptional activation and enzymatic output was noted for ticarcillin and carbenicillin.^30–32^ These compounds predominantly target PBP3, a non-essential high-molecular-weight DD-transpeptidase involved in septal peptidoglycan synthesis, whose inhibition does not generate the muropeptide signals typically associated with LMW-PBPs such as PBP4.^10,11,20^ Consequently, PBP3 inhibition fails to activate the CreBC two-component system, which has been implicated in the full induction of *ampC* and its post-transcriptional regulation.^2,10,11,20,33^ This mechanistic divergence accounts for the increased propensity of third-generation cephalosporins and aztreonam to select for constitutively derepressed AmpC mutants, as opposed to agents that primarily target LMW-PBPs.^29,31,34,35^

In contrast, cefepime combines minimal inducibility with certain structural resilience to *P. aeruginosa* AmpC-mediated hydrolysis, owing to the formation of a stable acyl-enzyme complex that significantly reduces catalytic turnover. Indeed, only approximately 5%–10% of AmpC-producing strains—typically those exhibiting both marked derepression and efflux upregulation—display clinical resistance to this compound.^12,22,36,37^

Among β-lactamase inhibitors, only clavulanic acid and avibactam raised *ampC* mRNA, and this transcriptional signal did not translate into increased enzyme output.^17,38,39^ Avibactam, a diazabicyclooctane inhibitor, binds reversibly to AmpC with a *K*d of ∼0.5 μM and a deacylation half-life of ∼6 min, enabling dynamic equilibrium and frequent rebinding.^40,41^ This reversible acylation masks hydrolytic activity *in vitro*. Furthermore, clavulanic acid did not induce *ampC* activity in our assays using concentrations of ≤4 mg/L (clinically and mechanistically relevant range), mirroring early reports that showed significant β-lactamase activity induction only at above-physiological peak concentrations of 8–50 mg/L.^38,39,42^

These findings underscore the pivotal role of PBP4 in *ampC* induction, while highlighting that its inhibition alone may be insufficient for full derepression. Although *ampC* transcriptional responses at PBP4_30_IC_50_ concentrations remained low for most of the compounds, strong enzymatic activity was still observed with imipenem and cefoxitin. This suggests that additional factors beyond PBP4 binding, such as PBP cooperativity or post-transcriptional regulation, may shape AmpC activity.^22,43^ Notably, while *ampC* mRNA levels and β-lactamase activity are positively correlated, the relationship is not linear: even modest increases in transcription can result in disproportionately high enzymatic activity and MIC values. This non-linear dynamic suggests that β-lactamase regulation is finely tuned and highly sensitive to subtle shifts in transcriptional input, possibly due to through enzyme accumulation or enhanced catalytic turnover.^31,44,45^

To confirm and further elucidate the central role of LMW-PBPs in *ampC* regulation in *P. aeruginosa*, we quantified *ampC* mRNA expression and β-lactamase activity in isogenic mutants with sequential deletions of LMW-PBPs. Stepwise inactivation of the LMW-PBP-encoding genes *dacB*, *dacC*, and *pbpG* resulted in a progressive increase in *ampC* mRNA levels, consistent with previous findings linking the loss of LMW-PBPs to muropeptide accumulation and constitutive *ampC* derepression via AmpR.^2,10–12^ Notably, despite transcript levels rising by up to ∼7000-fold, the corresponding increase in β-lactamase activity was comparatively modest (up to ∼1100-fold, or 1521 pmol nitrocefin min^−1^ mg^−1^ of protein), suggesting the existence of a post-transcriptional or post-translational bottleneck.^12,46^ The graded increase in *ampC* transcription observed under ceftazidime treatment across the LMW-PBP mutants can be attributed, at least in part, to the use of 0.5 × MIC relative to each strain, resulting in higher concentration and effective induction in the triple mutant. The absence of detectable β-lactamase activity following ceftazidime exposure, together with unaltered periplasmic and extracellular enzyme levels, further supports the notion of differential regulation or delayed enzyme maturation in this context.^10,12,35,46^

Furthermore, canonical inducers such as imipenem and cefoxitin elicited modest or even negligible transcriptional responses in LMW-PBP (derepressed *ampC*) mutants. Notably, in the complete absence of LMW-PBPs (PAOΔ*dacBdacCpbpG*), ceftazidime was the only compound capable of triggering a transcriptional response, further reinforcing the concept of differential *ampC* induction via PBP3 inhibition.^29,37^ Nevertheless, consistent with earlier data in PAO1, ceftazidime exposure failed to elicit any appreciable increase in β-lactamase activity, highlighting a persistent dissociation between *ampC* mRNA upregulation and enzymatic output for this compound. This may reflect a delayed or reduced production of active AmpC enzyme, as illustrated by the moderate ∼6-fold increase in PAO1 after 6 h of ceftazidime and aztreonam treatment and the absence of a significant rise in the triple mutant PAOΔ*dacBdacCpbpG* (data not shown).^29,46^ These findings underscore that *ampC* transcriptional activation does not inherently predict functional enzyme production, with post-transcriptional and translational (fitness-preserving) constraints likely playing a key regulatory role.^8,15^

A precise characterization of *ampC* induction—integrating PBP-binding affinities (IC_50_), transcriptional responses, and enzymatic activity—is essential for informing therapeutic strategies against *P. aeruginosa*. These findings have direct clinical implications, particularly in the context of rational antibiotic selection and the design of novel combination therapies involving β-lactamase inhibitors, where the interplay between induction and inhibition must be carefully assessed. The present findings establish a framework for future investigations into the regulatory and structural determinants of alternative *ampC* control and their role in shaping β-lactam resistance phenotypes.

## Supplementary Material

dkaf408_Supplementary_Data

## Supplementary data

Figures S1 and S2 and Tables S1–S4 are available as Supplementary data at *JAC* Online.
