# Supplementary material for: Unravelling the triad of penicillin-binding proteins, β-lactamase activity, and mRNA dynamics in Pseudomonas aeruginosa AmpC induction
Source: J Antimicrob Chemother. 2025 Nov 5;81(1):dkaf408. doi: 10.1093/jac/dkaf408 (PMC12802951; doi:10.1093/jac/dkaf408)
Supplement: dkaf408_Supplementary_Data [file dkaf408_supplementary_data.zip › JAC_PA_Induction_Supplementary_Material_R2_clean.docx]

**Supplementary data for:**

Montaner et al; **Unraveling the Triad of Penicillin-Binding Proteins, Beta-Lactamase Activity, and mRNA Dynamics in *Pseudomonas aeruginosa* AmpC Induction.**

^a^


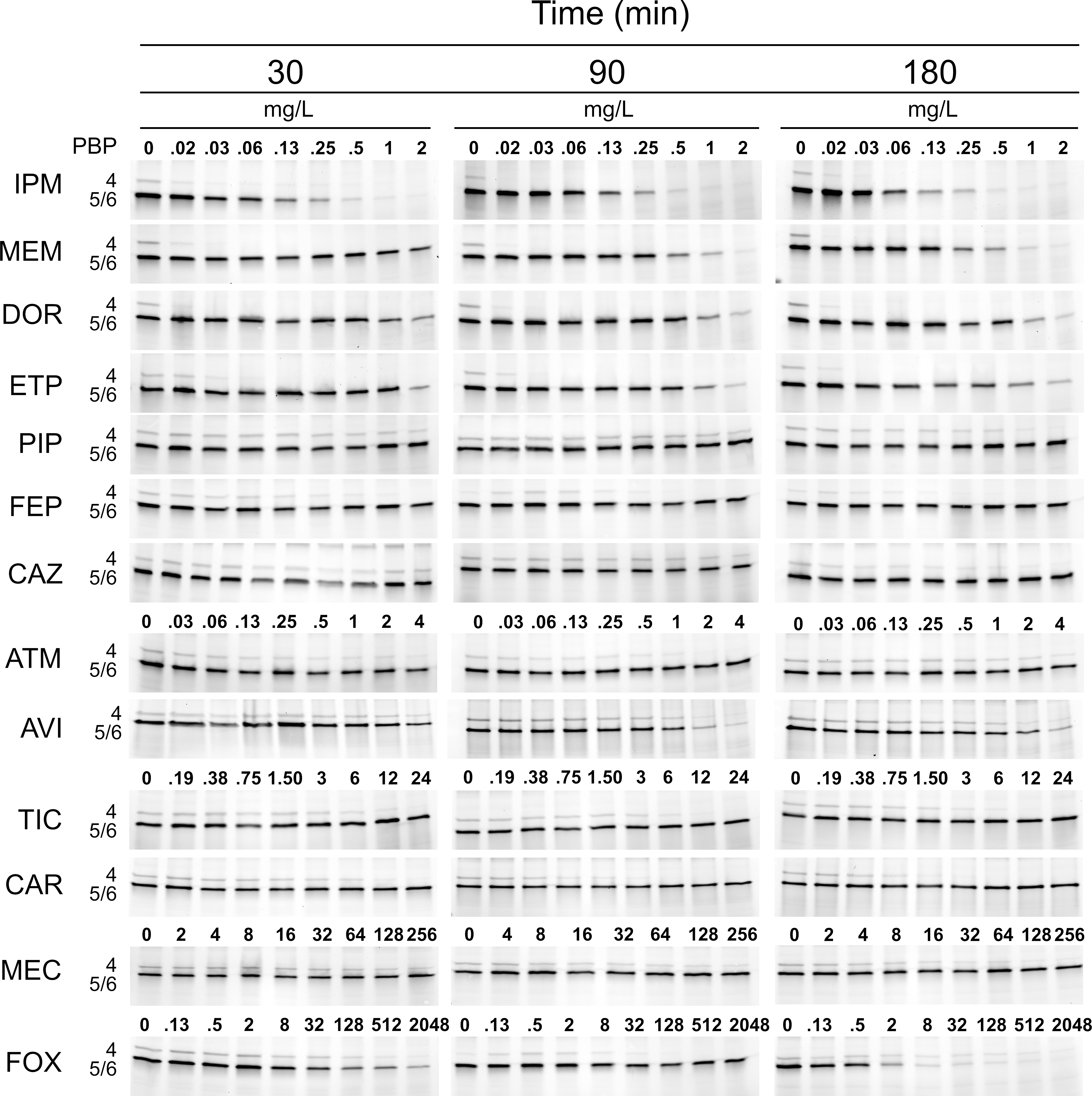


**Fig S1.** Penicillin-binding protein occupancy dataset binding affinity of PBP4 and PBP5 in intact PAO1 cells at intervals of 30, 90, and 180 minutes following incubation with a panel of 12 structurally diverse β-lactams, which included 4 carbapenems, 3 cephalosporins, 3 penicillins, and 1 monobactam, alongside 6 β-lactamase inhibitors (BLIs). Ranges of concentration used are depicted in each subset of drugs.

^a^ Drugs tested were: IPM, imipenem; ETP, ertapenem; MEM, meropenem; DOR, doripenem; PIP, piperacillin; TIC, ticarcillin; CAR, carbenicillin; MEC, mecillinam; ATM, aztreonam; FEP, cefepime; CAZ, ceftazidime; FOX, cefoxitin and AVI, avibactam. The antibiotic-bound PBP-containing membrane preparations were labeled with 25 μM Bocillin FL. Labeled PBPs were separated by SDS-PAGE and detected using a fluorimager. The global range of concentrations tested was 0.002 to 2048 mg/L.


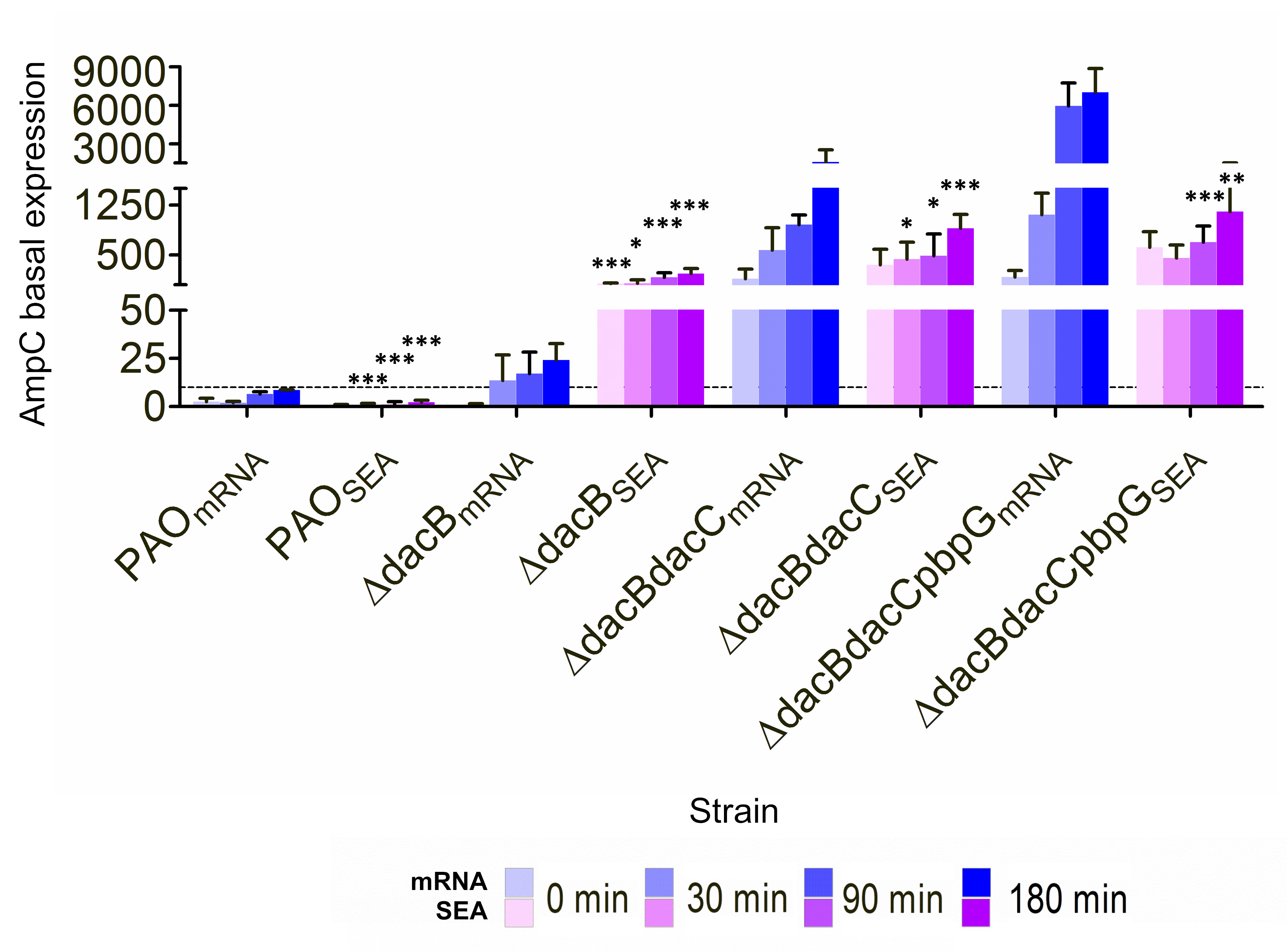


**Figure S2**. The relative expression (relative to PAO1 at t = 0 min) of *ampC* (mRNA) in in *P. aeruginosa* strains PAO1 and isogenic mutants PAOΔ*dacB*, PAOΔ*dacBdacC* and PAOΔ*dacBdacCpbpG*, as measured by qRT-PCR, and the corresponding relative specific enzymatic activity (SEA) were assessed at time points of 0, 30, and 180 minutes. Results are averages of at least two sets (biological replicates) of two technical replicates. Unpaired Student’s t tests were applied to determine statistically significant differences between mRNA and SEA determinations (*, P-value < 0.05; **, P-value < 0.01; ***, P-value < 0.005).

**Table S1.** One-way ANOVA with post hoc Tukey’s multiple comparison test was run to determine the statistically significant differences over time (*, P-value < 0.05; **, P-value < 0.01; ***, P-value < 0.005).

**Table S2**. PBP binding (IC_50_) of β-lactam antibiotics and BLIs in lysed *P. aeruginosa* PAO1.

| PBP |  | IC_50_ of the indicated drug (mg/L)*^a^* | | | | | | | | | |
| --- | --- | --- | --- | --- | --- | --- | --- | --- | --- | --- | --- |
|  | IPM*^b^* | DOR | MEM*^c^* | ETP | FOX | CAZ | FEP*^c^* | ATM | MEC | PIP | AVI*^b^* |
| 1a | 0.19 | 0.43 | 0.26 | 0.35 | 7.8 | **0.16** | **0.12** | 2.11 | >4 | 1.57 | >32 |
| 1b | 0.13 | 0.37 | 0.21 | 0.36 | >32 | >4 | 0.82 | 2.95 | >4 | 1.11 | 3.6 |
| 2 | **0.05** | **0.06** | **0.13** | **0.04** | 14.6 | >4 | 2.71 | >4 | **0.19** | 3.17 | 4.2 |
| 3 | 0.34 | **0.13** | **0.06** | **0.19** | 10.7 | **0.06** | **0.05** | **0.08** | >4 | **0.05** | >32 |
| 4 | **0.01** | **0.01** | **0.01** | **0.02** | **1.5** | 2.15 | 2.52 | >4 | >4 | 1.45 | 3.1 |
| 5/6 | 0.52 | 1.41 | 0.31 | 2.21 | 9 | >4 | >4 | >4 | >4 | >4 | 2.2 |

*^a^* Concentration of β-lactam that inhibits 50 % of Bocillin FL compared to no drug control. IPM, imipenem; DOR, doripenem; MEM, meropenem; ETP, ertapenem; FOX, cefoxitin; CAZ, ceftazidime; FEP, cefepime; ATM, aztreonam; MEC, mecillinam; PIP, piperacillin; AVI, avibactam. ^1^ Bold numbers represent the selective PBPs for a given compound (i.e. IC_50_ ≥ 4-fold lower than that of the next most inhibited PBP). ^2^

*^b^* IPM and AVI IC_50_ values reproduced from our previous work Lopez-Argüello et al, 2021. ^3^

*^c^* MEM and FEP IC_50_ values reproduced from our previous work Moya et al, 2017. ^4^

**Table S3**. Comparison of the specific enzymatic activity (SEA) using nitrocefin and cefalotin as substrates.

| Drug  (concentration) | SEA*^a^* | | | | | | | |
| --- | --- | --- | --- | --- | --- | --- | --- | --- |
|  | Time (min) | | | | | | | |
|  | 0 | | 30 | | 90 | | 180 | |
|  | NTC | CEF | NTC | CEF | NTC | CEF | NTC | CEF |
| Ctrl (0) | 4.12 | 5.95 | 2.63 | 3.21 | 1.89 | 2.17 | 3.51 | 2.98 |
| IPM (0.5 × MIC) | 1.55 | 2.31 | 998.3 | 767.5 | 1056.4 | 867.4 | 708.1 | 938.7 |
| FOX (0.5 × MIC) | 2.13 | 4.80 | 432.6 | 328.9 | 1482.6 | 1125.8 | 966.7 | 1042.3 |
| TIC (0.5 × MIC) | 1.17 | 3.32 | 0.72 | 3.54 | 1.04 | 4.15 | 3.72 | 5.56 |
| IPM (IC_50_PBP4_30’_) | 2.62 | 3.08 | 537.4 | 358.1 | 111.5 | 199.9 | 20.83 | 32.27 |
| FOX (IC_50_PBP4_30’_) | 1.25 | 3.62 | 22.15 | 36.55 | 59.98 | 103.4 | 36.34 | 43.90 |
| TIC (IC_50_PBP4_30’_) | 1.11 | 2.61 | 4.46 | 2.70 | 1.07 | 0.85 | 16.24 | 9.95 |

*^a^* Specific enzymatic activity (SEA) using nitrocefin (NTC) or cefalotin (CEF) as a substrate, following exposure to 0.5 times the minimum inhibitory concentration (0.5 × MIC) and the concentration that half maximally inhibits PBP4 after 30 mins (IC_50_PBP4_30’_) of imipenem (IPM), cefoxitin (FOX) and ticarcillin (TIC) at time points of 0, 30, and 180 minutes. Results are averages of at least two sets (biological replicates) of two technical replicates.

**Table S4.** Inhibition of AmpC activity by 50 mg/L cloxacillin in the triple PAΔ*dacBdacCpbpG* PAO1 mutant with constitutive hyperexpression.

| Drug  (concentration) | SEA*^a^* | | | | | | | |
| --- | --- | --- | --- | --- | --- | --- | --- | --- |
|  | Time (min) | | | | | | | |
|  | 0 | | 30 | | 90 | | 180 | |
|  | NTC | CEF | NTC | CEF | NTC | CEF | NTC | CEF |
| Ctrl (0) | 615.2 | 475.2 | 552.4 | 497.4 | 693.2 | 649.3 | 1154.2 | 860.2 |
| CLX (50 mg/L) | 6.88 | 9.56 | 9.64 | 6.87 | 5.56 | 5.27 | 11.88 | 9.25 |
| CLX (25 mg/L) | 12.22 | 11.21 | 10.62 | 12.62 | 8.45 | 9.84 | 14.35 | 18.47 |

*^a^* Specific enzymatic activity (SEA) of the in the triple PAΔ*dacBdacCpbpG* PAO1 mutant under basal conditions (Ctrl) and after 3 minutes pre-incubation with 500 mg/L cloxacillin (CLX), using nitrocefin (NTC) or cefalotin (CEF) as a substrate. Results are averages of at least two sets (biological replicates) of two technical replicates. A reduction > 95% in nitrocefin or cefalotin hydrolysis was considered indicative of effective β-lactamase activity inhibition by cloxacillin.

**REFERENCES**

1. Montaner, M., et al., PBP Target Profiling by β-Lactam and β-Lactamase Inhibitors in Intact *Pseudomonas aeruginosa*: Effects of the Intrinsic and Acquired Resistance Determinants on the Periplasmic Drug Availability. Microbiol Spectr, 2023. 11(1): p. e0303822.

2. Kocaoglu, O. and E.E. Carlson, Profiling of beta-lactam selectivity for penicillin-binding proteins in *Escherichia coli* strain DC2. Antimicrob Agents Chemother, 2015. 59(5): p. 2785-90.

3. López-Argüello, S., et al., Molecular Basis of AmpC β-Lactamase Induction by Avibactam in *Pseudomonas aeruginosa*: PBP Occupancy, Live Cell Binding Dynamics and Impact on Resistant Clinical Isolates Harboring PDC-X Variants. Int J Mol Sci, 2021. 22(4): p. 3051.

4. Moya, B., et al., WCK 5107 (Zidebactam) and WCK 5153 Are Novel Inhibitors of PBP2 Showing Potent "β-Lactam Enhancer" Activity against *Pseudomonas aeruginosa*, Including Multidrug-Resistant Metallo-β-Lactamase-Producing High-Risk Clones. Antimicrob Agents Chemother, 2017. 61(6).
